# Supplementary material for: Association between oral microbiome and seven types of cancers in East Asian population: a two-sample Mendelian randomization analysis
Source: Front Mol Biosci. 2023 Nov 21;10:1327893. doi: 10.3389/fmolb.2023.1327893 (PMC10702768; doi:10.3389/fmolb.2023.1327893)
Supplement: Supplementary file 1 [file DataSheet1.ZIP › Supplementary Materials/MR plots for tongue/tongue═╝/Gastric cancer/pheno.243_to_gastric cancer_leave_one_out.pdf]

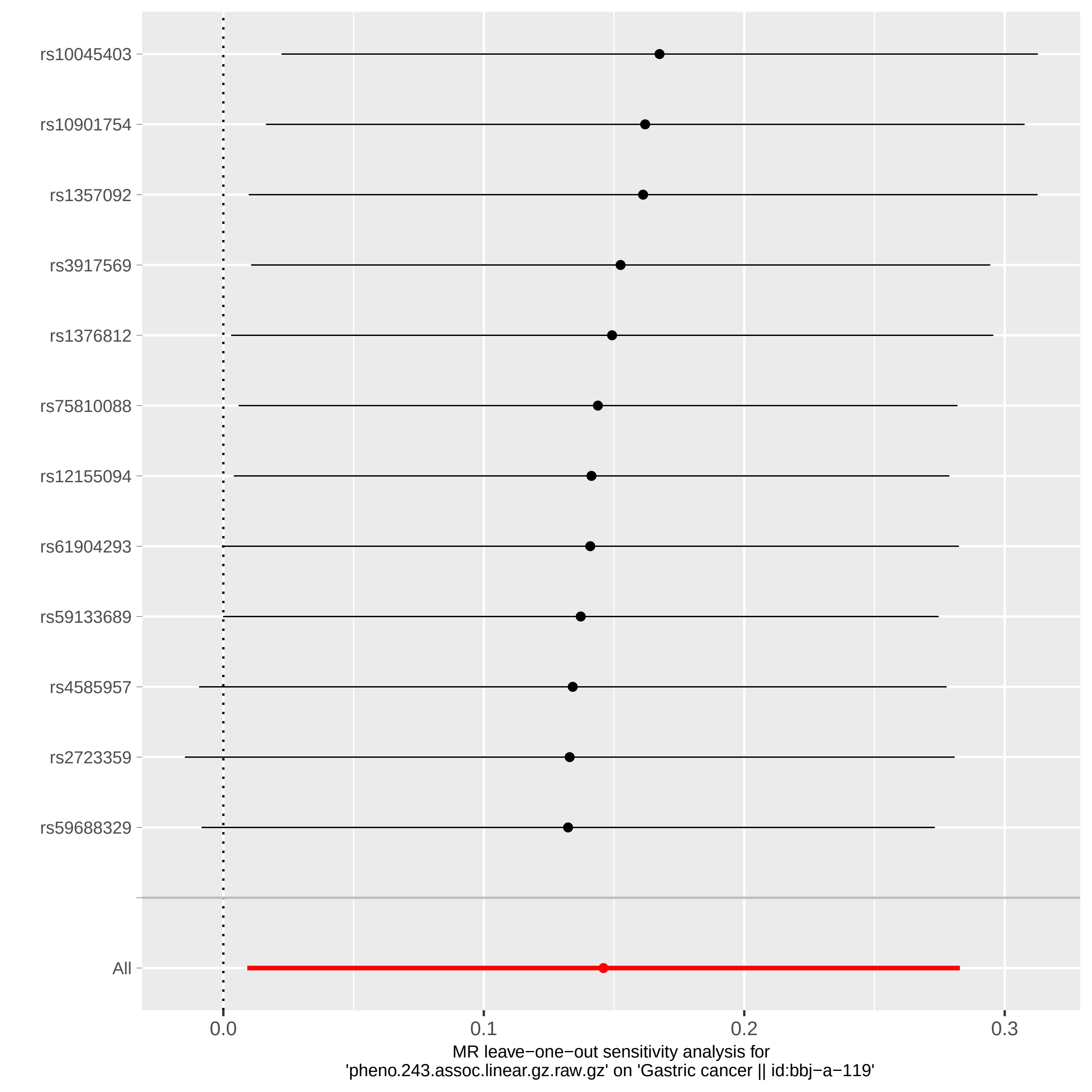
rs10045403
rs10901754
rs1357092
rs3917569
rs1376812
rs75810088
rs12155094
rs61904293
rs59133689
rs4585957
rs2723359
rs59688329
All
0.0
0.1
0.2
0.3
MR leave−one−out sensitivity analysis for
'pheno.243.assoc.linear.gz.raw.gz' on 'Gastric cancer || id:bbj−a−119'
